# May Nutritional Status Positively Affect Disease Progression and Prognosis in Patients with Esophageal and Pharyngeal Cancers? A Scoping Review of the Current Clinical Studies

**DOI:** 10.3390/medsci11040064

**Published:** 2023-10-02

**Authors:** Georgios Antasouras, Sousana K. Papadopoulou, Maria Tolia, Aimilia-Lynn Pandi, Maria Spanoudaki, Nikolaos Tsoukalas, Gerasimos Tsourouflis, Evmorfia Psara, Maria Mentzelou, Constantinos Giaginis

**Affiliations:** 1 Department of Food Science and Nutrition, School of the Environment, University of the Aegean, 81400 Lemnos, Greece; g.antasouras@gmail.com (G.A.); fnsm22020@fns.aegean.gr (A.-L.P.); fnsd21013@fns.aegean.gr (E.P.); maria.mentzelou@hotmail.com (M.M.); 2 Department of Nutritional Sciences and Dietetics, Faculty of Health Sciences, International Hellenic University, 57400 Thessaloniki, Greece; souzpapa@gmail.com (S.K.P.); maryspan1@gmail.com (M.S.); 3 Department of Radiotherapy, Faculty of Medicine, School of Health Sciences, University of Crete, 71110 Heraklion, Crete, Greece; mariatolia1@gmail.com; 4 Department of Oncology, 401 General Army Hospital of Athens (401 Geniko Stratiotiko Nosokomeio Athenon), 11525 Athens, Greece; tsoukn@yahoo.gr; 5 Second Department of Propedeutic Surgery, Medical School, University of Athens, 11527 Athens, Greece; gtsourouflis@med.uoa.gr

**Keywords:** nutritional assessment, esophageal cancer, pharyngeal cancer, prognosis, survival, nutritional status, diagnosis, malnutrition, chemotherapy, radiotherapy

## Abstract

Background: Malnutrition in esophageal and pharyngeal cancer patients constitutes a common and serious concern, which significantly reduces patients’ prognoses. Cancers of the esophagus and the pharynx can considerably impair feeding in patients, resulting in severe undernutrition. This is a scoping review that intends to critically analyze the most well-designed clinical studies investigating the potential beneficial impact of diverse nutritional assessment tools on the prognosis of patients with esophageal and pharyngeal cancers. Methods: The most accurate and remarkable scientific databases were comprehensively explored utilizing relative keywords to detect clinical studies that investigate whether nutritional status may affect disease prognosis. Results: Several assessment tools have evaluated and highlighted the potential beneficial impact of nutritional status on disease progression and patients’ prognosis in both esophageal and pharyngeal cancers. Regarding esophageal cancer, CONUT, PNI, PG-SGA, and NRS-2002 are more commonly used, while albumin is also frequently evaluated. Regarding pharyngeal cancers, fewer studies are currently available. PNI has been evaluated, and its significance as a factor for shorter survival’ times has been highlighted. The Comprehensive Nutritional Index has also been evaluated with positive results, as well as NRS 2002, GPS, and body-weight status. However, there is currently a lack of studies with an adequate number of women with cancer. An international literature gap was identified concerning follow-up studies with adequate methodology. Conclusions: Nutritional status may significantly affect disease progression and patients’ survival, highlighting the significance of a great nutritional status in individuals with esophageal and pharyngeal cancers. Further large-scale and well-designed prospective surveys should be performed to verify the potential beneficial effects of adequate nourishment in people suffering from cancer of the esophagus and pharynx.

## 1. Introduction

Cancer is one of the most common causes of death, accounting for one in six deaths worldwide, according to the World Health Organization (WHO) [1]. According to the International Agency for Research on Cancer (IARC), cancer of the esophagus accounted for 3.1% (604,100) of new cases, and 5.5% (544,076) of deaths worldwide in 2020 [1]. In the meantime, 98,412 new cases of oropharyngeal cancers, and 133,354 new cases of nasopharyngeal cancers were diagnosed, while 48,143 and 80,008 deaths were accounted for due to oropharyngeal and nasopharyngeal cancers, respectively [1]. According to global cancer statistics, esophageal cancer is higher in East Asia and South Africa, while the incidence is lower in Western and Central Africa and Central America, with a difference of nearly 12 times in incidence [2,3]. The incidence of esophageal adenocarcinoma has remarkably risen during the last decades; it was about three times higher in males than in females, while the 5 year overall survival rate for all types of esophageal cancer was only 18%. Meanwhile, esophageal adenocarcinoma has become the predominant type of esophageal cancer in Northern America and Northern Europe, accounting for nearly 60%, while esophageal squamous cell carcinoma accounts for about 34% [2,3]. Oral cavity and pharyngeal cancer collectively rank seventh for incidence and eighth for cancer mortality [4,5]. Oral and pharyngeal cancers are strongly associated with alcohol and tobacco consumption. Male death rates from oral and pharyngeal cancers steadily decreased since the mid-1980s in countries from southern Europe, such as France, Italy, and Spain, where male tobacco and alcohol consumption has long been declining [4,5]. Conversely, rates rose in more recent decades and reached exceedingly high values in men from several countries from Central and Eastern Europe, which had more unfavorable patterns in tobacco and alcohol use, particularly among men. Incidences of oral and pharyngeal cancer have been increasing over the last decade in the United States (USA), as well as a few other countries, mainly due to an increase in oropharyngeal cancer rates at a site associated with human papillomavirus (HPV) infection [4,5].

More than half of all cancer deaths are ascribed to lung, liver, stomach, colorectal, and breast cancers. Head and neck cancers, including esophageal and pharyngeal cancers, are also very common [6]. Around one third of cancer deaths are caused by the following lifestyle choices: low fruit and vegetable intake, and dietetic fibers, obesity, low levels of physical activity, heavy smoking, and alcohol overconsumption. Smoking has been considered the most important risk factor for cancer, causing 20% of cancer deaths and around 70% of lung cancer deaths worldwide [6,7]. Both genetic and environmental factors can considerably increase cancer risk and development. Moreover, hereditary genetic mutations (germline mutations) are nonmodifiable risk factors, which can be detected by genetic testing. Currently, it is estimated that merely about 2–3% of diagnosed cancers are associated with an inherited mutated gene [8]. Epigenetic mutations are the result of the interaction between a person’s genetic factors and three categories of external agents, including physical carcinogens (e.g., ultraviolet and ionizing radiation), chemical carcinogens (e.g., asbestos, components of tobacco smoke, aflatoxin, and arsenic), and biological carcinogens (infections from certain viruses, bacteria, or parasites) [7,8].

Malnourishment constitutes a frequent finding in patients with cancer, even at the time of diagnosis. Malnourishment prevalence ranges from 31% to 87% and depends on the tumor’s histopathological stage and type, medication, and the personalized characteristics of each patient [6,7]. Body weight decline commonly arises due to enhanced energetic requests, low energetic intake, and/or the existence of nutrient malabsorption. In carcinoma patients, undereating could be ascribed to several causes. Inflammation and catabolism related to tumor development and progression may also result in atrophy of muscles, underweight, and sarcopenia, while cancer gastrointestinal blockage may diminish food consumption and may lead to malabsorption. In particular, swallowing difficulties, aches, and nausea could be triggered [8,9]. In addition, cancer treatment may result in several adverse side effects, including low appetite, early satiety, nausea, sickness, oral and intestine mucositis with low swallowing disturbances, diarrhea, hemorrhoids, and anal fissures; alterations in smelling and tasting disturb not merely the whole energetic increase, but also the increased risk of nutrient malabsorption, negatively affecting nutritional status [9]. The presence of cognitive impairments in cancer patients is also able to influence their capability to take energy by food consumption [9].

Malnourishment in esophageal and pharyngeal cancers is a common and serious concern, with dysphagia severely impairing patients’ nutritional status [10]. Malnutrition also increases the probability of low compliance to medication therapy and radiotherapy and, finally, adverse disease outcomes [11,12,13]. Cancers of the esophagus and the pharynx can impair feeding in patients and lead to undernutrition [11,12,13]. Nutrition impact symptoms negatively influence cancer patients beyond the acute phase of cancer therapy [14,15,16]. These symptoms are linked with reduced nutrition and quality of life. Notably, 23.8–48.9% of patients with oral cancers are diagnosed with malnutrition [14,15], while the rates for esophageal cancer patients reach 79% [13].

Nutritional assessment is important in order to prevent and manage malnutrition [15], whilst nutritional status acts as a prognostic factor for disease progression [17]. A substantial concern regarding malnutrition is underdiagnosis [18], even if nutritional assessment testing is recommended to be accomplished during the diagnostic procedure [16]. Moreover, analyzing body composition can decrease the likelihood of medication toxicity, can be favorable for prognosis, reduce disease development, decrease the risk of complications due to surgery, improve performance status, and increase survival times [19]. In contrast, BMI alone cannot differentiate fat mass from fat-free mass and is not representative of body-weight decline [20].

In this context, the purpose of this scoping review is to effectively analyze and scrutinize the existing clinical evidence as far as the use of diverse nutritional screening questionnaires as concerns on the disease development and prognosis of individuals with esophageal and pharyngeal cancers.

## 2. Methods

This is a scoping review that intends to summarize in depth the currently available scientific data concerning the impact of nourishing state in disorder development and survival of patients with esophageal or pharyngeal cancers. Moreover, it aims to find the most important and reliable data and the lack of international scientific literature as well as the key concepts and the original information to notify new approaches, policies, and strategies concerning this specific scientific area. Only clinical studies in humans, which were published between the period 2000–2023 and which were written in the English language were included. The included clinical studies should be published only in peer-reviewed scientific journals, and they should investigate a measure for the burden of treatment.

This scoping review includes only clinical studies that applied quantitative and qualitative methodologies or even mixed methodologies to explore diverse approaches to determining treatment burden. A thorough investigation of the currently available evidence was performed in the most reliable scientific databases, e.g., PubMed, Scopus, Web of Science, and Google Scholar, using efficient, representative, and relevant keywords, such as nutrition, nutritional assessment, nutritional status, nutritional tools, cancer, esophageal cancer, pharyngeal cancer, cancer progression, patients’ prognosis, survival, clinical studies, etc. We also searched the references of relevant review article commentaries, editorials, and abstracts in congresses’ proceedings. The recovered studies were also systematically tested for relevant papers reported in their text.

The collected papers were reviewed by all authors. To enhance reliability between the reviewers, all reviewers comprehensively read all the recovered papers, discussed the findings, revised the initial findings, and extracted the evidence manual prior to the start of screening for this review. Six reviewers cooperating in groups of two and in sequence assessed the titles, abstracts, and, subsequently, the full text of all published papers to, through our investigations, detect articles probably related. When there was no agreement on study selection and the extracted evidence, all the authors/reviewers discussed together to resolve their different opinions, if required. 

A data mapping document was cooperatively derived by two reviewers (S.K.P. and C.G.) to decide the appropriate variables that should be obtained. Each of the two reviewers individually monitored the evidence, reviewed the findings, and constantly reorganized the data-charting document in a repetitive procedure. When we found a systematic review, we analyzed its included surveys, which probably agreed with our inclusion standards and noticed how many surveys had not been found by our research. We included merely follow-up, cross-sectional, descriptive, pilot, or case-report clinical studies. In vitro and in vivo animal studies were not included. Articles were not included if they were not in line with the theoretical basis of the study. We included only papers evaluating the effect of nourishment state on disease development and survival of individuals diagnosed with cancers in the esophagus or pharynx.

**Figure 1:**
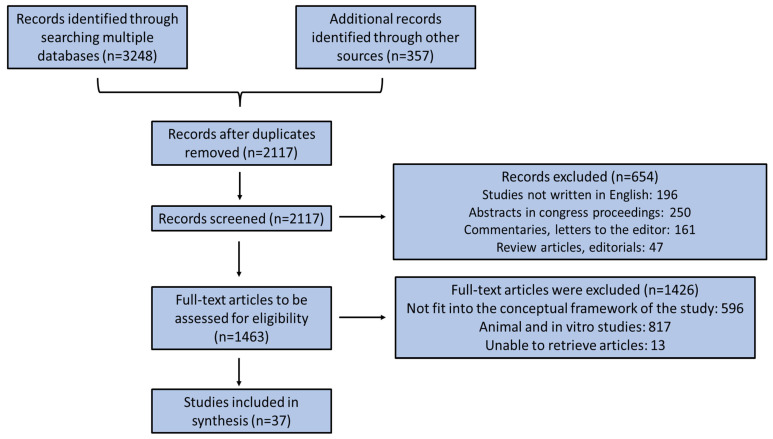
Flow chart diagram of the enrolled studies.

Clinical studies that included individuals with other tumor malignancies simultaneously in other organs of the human body beyond the esophagus and pharynx were not included. The results were chosen according to their relevance, and the most relevant ones were selected and stated underneath based on the flowchart diagram, as shown in Figure 1. We scrutinized emerging findings concerning whether a good nutritional status may ameliorate patients’ prognosis and disease symptomatology, simultaneously slowing down disease development, to contribute to the mapping of the literature on this exact issue, which could help forthcoming research and systematic reviews on this topic.

## 3. Results and Discussion

### 3.1. Esophageal Cancer

Esophageal cancer is associated with shorter survival times due to the increased incidence of malnourishment and cachexia in patients with the disease [21], which is prevalent in patients with obesity, as well [22]. Although the prognostic significance of the nutritional status does not mean exclusion from treatment [23], the need for timely nutritional assessment is important, as interventions for the treatment of malnutrition can considerably improve survival [24]. There are adequate, validated questionnaires that evaluate nourishing state, which have been applied in several studies, and shown prognostic potential [25,26]. In fact, Wang et al. performed a prospective study in 192 patients with esophageal carcinomas and found that both the Geriatric Nutritional Risk Index (GNRI) < 92 and the European Society of Clinical Nutrition and Metabolism (ESPEN 2015) 2015, two malnutrition diagnosis reference tools, showed good property in predicting major complications, infectious complications, overall complications, and delayed hospital discharge [25]. By performing a retrospective analysis of 155 esophageal cancer patients, they also confirmed the better performance of GNRI < 92 in predicting perioperative morbidities than the other three nutritional indexes [25]. In a retrospective study, 340 esophageal squamous cell carcinoma patients who completed curative treatment and received a nutrition evaluation by the Patient-Generated Subjective Global Assessment (PGSGA) score; malnutrition (patients with a high PGSGA score) was associated with advanced stage and reduced survival rate [26]. Surgical resection brought the survival benefit to patients in the low PGSGA group, but not for the malnourished patients after neoadjuvant treatment [26]. Table 1 includes all clinical studies evaluating the use of the prognostic role of diverse nutritional assessment tools on the survival of patients with esophageal carcinoma.

A plethora of recent surveys have explored the potential importance of “Controlling Nutritional Status” (CONUT) scoring in patients’ survival of esophageal carcinoma. These studies showed that CONUT scoring may be a significant prognosticator of overall and disease-specific survival in patients who have undergone esophagectomy [27]. More to the point, a systematic literature review was carried out to investigate the impact of the CONUT score in esophageal cancer, including five studies with 952 patients [27]. This meta-analysis found a significant association of the CONUT score with outcomes including overall survival, cancer-specific survival, and recurrence-free survival [27]. Moreover, in a recent retrospective survey of 69 individuals with progressed esophageal cancer receiving treatment with an immune checkpoint suppressor, the CONUT score was independently associated with overall and progression-free patient survival [28]. In addition, among patients treated with an immune checkpoint inhibitor, a high CONUT score was associated with significantly worse progression-free survival (PFS) and overall survival compared with a low CONUT group [28]. A retrospective survey by Hirahara et al. (2018) conducted on 148 consecutive patients who underwent potentially curative surgery for histologically verified esophageal squamous cell carcinoma also confirmed that the CONUT scoring was independently associated with cancer-specific survival in patients aged < 70 years old who undergo curative surgery [29]. Further retrospective studies highlight that the CONUT score can predict malnutrition and act as a prognosticator of overall and disease-specific survival in patients treated with surgery [30,31]. A retrospective study was performed on 352 patients who had undergone elective esophagectomy with lymphadenectomy for esophageal cancer and were assigned to three groupings based on the CONUT assessment [30]. Malnourished patients exhibited a considerably elevated prevalence of any morbidity, serious morbidities, and surgical site infections. Hospitalization of malnourished patients was found substantially extended. In a multivariate analysis, intermediate or advanced malnourishment was independently associated with the probability of any morbidity and serious morbidities [30]. Another retrospective study by the same research group was conducted on 373 patients who had undergone three-incision esophagectomy with two- or three-field lymphadenectomies due to esophageal carcinoma [31]. This study showed that malnourished patients underwent a considerably elevated incidence of reoperation and a greater tendency of lung morbidities [31]. CONUT score was able to predict malnutrition and acted as a prognosticator of overall and disease-specific patient survival [31]. Regarding recurrence, a recent retrospective study using the CONUT score, with a cut-off point of three, found that patients who underwent neoadjuvant immunochemotherapy with high CONUT were more likely to relapse, while those with a reduced CONUT scoring exhibited a favorable disease-free survival after one year [32]. Moreover, vessel invasion, postoperative pneumonia, and advanced ypT, cTNM, and ypTNM stages were substantially related to patients scoring high CONUT values [32].

The Prognostic Nutritional Index (PNI) score has been applied in a few recent studies with esophageal carcinoma patients. In fact, PNI was used in a study group of 337 patients, and those with a low PNI (<45) had shorter overall survival than those with a high PNI score [33]. Interestingly, PNI was considerably related to tumor-infiltrating lymphocytes (TILs) state and CD8-positive cell count, supporting evidence that nutritional status and systemic immune competency could affect patient survival via local immune response [33]. A recent study on 407 patients who underwent curative esophagectomy indicated that a reduced PNI score < 48.33 was independently associated with overall survival, being also associated with a high prevalence of postoperative complications [34]. A smaller study with 32 individuals with esophageal squamous cell cancer who experienced salvage esophagectomy showed that PNI, with a cut-off point of 45, was identified as an independent preoperative prognosticator for overall survival [35]. After adjustment for patient age, clinical response, and preoperative PNI, PNI was not a prognosticator for disease-specific survival [35]. Nakatani et al. (2017) investigated the potential of PNI to predict prognosis in 66 individuals who had undergone neoadjuvant chemotherapy and applied as a cut-off point the PNI score of 45, like the aforementioned studies [36]. Preoperative PNI was independently associated with shorter overall and relapse-free survival, yet prechemotherapy PNI was not independently correlated with overall and relapse-free survival [36]. Wang et al. (2018) showed that PNI was independently associated with overall survival, but not with progression-free survival, organ metastasis-free survival, and local regional relapse-free survival [37]. Concerning patients at risk of malnutrition, the average days of patients staying in the hospital for nutritional support were significantly shorter, and the mean costs of staying in the hospital were smaller than those without nourishing care [37].

The Patient-Generated Subjective Global Assessment (PG-SGA) is another diagnostic questionnaire that has been explored in individuals with esophageal cancer. Movahed et al. (2020) followed up 71 newly diagnosed patients for one year [38]. At the end of the first year, mortality was related to decreased BMI prior to chemoradiotherapy, baseline PG-SGA scoring, weight decline, low BMI (<18.5 kg/m^2^), and decreased mid-upper arm circumference (MUAC) [38]. The twelve-month mortality was significantly associated with lower BMI after chemoradiotherapy, primary PG-SGA score, weight loss, BMI < 18.5, MUAC, physical performance, living in rural or urban areas, and addiction [38]. Furthermore, Chen et al. (2021) performed a study on 620 newly diagnosed patients with esophageal squamous cell cancer at stages T2 to T4 or regional lymph node metastasis. This study used five nutritional parameters: serum albumin, (BM), GNRI, prognostic nutritional index (PNI), and a new modified nutritional risk index (mNRI). All nutritional parameters were significantly correlated with tumor length and pT category. Decreased nutritional parameters were significantly correlated with poor survival in univariate analysis; however, only the mNRI was an independent prognostic factor in multivariate analysis [39]. A meta-analysis study including 15 studies and enrolling 1864 participants found that preoperative nutrition could reduce infectious complications and length of hospital stay after esophagectomy, whereas no significant difference was revealed in the incidence of overall complications in-hospital mortality, and anastomotic leak [40]. This study supported evidence that preoperative nutrition is safe in esophageal cancer; however, potential benefits can be observed in infectious complication rate and length of stay on a limited scale [40]. It is important to note that cut-off points may differ in different studies, which may confuse clinicians [38,39,40].

**Table 1:** Studies regarding esophageal carcinoma and the prognostic role of nourishment state.

Characteristics of Individuals with Esophageal Carcinoma	Assessment Tool	Results	Author, Date
258 patients randomly assigned to definitive chemoradiotherapy (dCRT) +/− cetuximab	NRI	Baseline NRI < 100 predicted worse overall survival.	Cox 2016 [24]
Retrospective study on 69 advanced esophageal carcinoma patients, aged 18–80 years, treated with Immune Checkpoint Inhibitor (ICI)	CONUT	CONUT score (cut-off point = 1) was an independent prognostic factor for overall survival and progression-free survival in patients undergoing ICI.	Chang 2022 [28]
Retrospective study of 148 patients with esophageal squamous cell carcinoma who underwent potentially curative esophagectomy (complete resection)	CONUT	CONUT scoring was independently associated with cancer-specific survival in patients aged <70 years old.	Hirahara 2018 [29]
Retrospective study of 352 patients who underwent elective esophagectomy with lymphadenectomy for esophageal carcinoma	CONUT	Malnourished patients had a substantially greater prevalence of any morbidity and surgical site infection. Hospitalization of malnourished patients was considerably longer. Intermediate or advanced malnutrition was independently associated with increased risk of any morbidity and serious morbidities.	Yoshida 2016 [30]
Retrospective study of 373 patients who underwent three-incision esophagectomy with 2- or 3-field lymphadenectomy for esophageal carcinoma	CONUT	CONUT score was able to predict malnutrition and acted as a prognosticator of overall and disease-specific survival in patients undergone surgery.	Yoshida 2017 [31]
216 patients with esophageal squamous cell carcinoma, receiving neoadjuvant immunochemotherapy	CONUT	↑ CONUT score (cut-off point = 3): ↑ risk of relapse. CONUT score: independent prognosticator for disease-free survival at one year.	Feng 2022 [32]
Database of 337 curatively resected esophageal cancers	PNI	↓ PNI led to considerably poorer overall survival in both univariate and multivariate analyses.	Okadome 2020 [33]
Retrospective study of 407 esophageal carcinoma patients who underwent esophagectomy	PNI	Multivariable analysis identified PNI as an independent prognosticator for overall survival and postoperative complications.	Qi 2021 [34]
Retrospective study with 32 patients with esophageal squamous cell carcinoma who underwent salvage esophagectomy	PNI	PNI (cut-off point = 45) was independently associated with overall survival preoperatively after adjustment for age, clinical response, and preoperative PNI. PNI was not a prognosticator for disease-specific survival.	Sakai 2018 [35]
66 squamous cell esophageal carcinoma patients undergoing neoadjuvant chemotherapy	PNI	The mean values of PNI score preoperatively and before treatment were 48.1 ± 4.7 and 50.2 ± 5.7, respectively. PNI decreased following chemotherapy in 66.7% of patients. Prechemotherapy PNI and PNI preoperatively were considerably correlated with the overall survival and relapse-free survival times. Only preoperative PNI was independently associated with worse overall and relapse-free survival.	Nakatani 2017 [36]
97 esophageal carcinoma patients earlier cured with definitive chemoradiotherapy (CRT)	PNI	PNI at diagnosis or PNI close to the ending of CRT (≥45) was related to better 2-year overall survival. PNI was a prognosticator for overall, but not for progression-free survival, organ metastasis-free survival, or local regional recurrence-free survival post-CRT.	Wang 2018 [37]
71 newly diagnosed patients followed for 1 year	PG-SGA	1 year mortality was considerably related to reduced BMI next to CRT, primary PG-SGA score, weight loss, BMI < 18.5, MUAC, physical performance, living in rural or urban areas, and addiction.	Movahed 2020 [38]
Retrospective study on 340 esophageal squamous cell carcinoma patients who completed curative treatment	PG-SGA	Well-nourished patients benefited from surgery. Malnutrition was associated with worse prognosis, regarding metastases and survival.	Chen 2021 [39]
202 patients with unresectable locally advanced esophageal carcinoma (stages 3 and 4) who were treated with definitive concurrent chemoradiotherapy	NRS-2002	NRS-2002 score (cut-off point ≥ 3) (was an independent prognosticator for the response to chemoradiotherapy, overall survival, and progression-free survival.	Song 2017 [41]
274 patients (stages 1 to 3, median age 63 years) undergone direct surgery for esophageal squamous cell carcinoma, with a median follow-up of 55 months	NRS-2002	The NRS 2002 group with elevated scores had shorter overall survival times. Elevated NRS 2002 scores were more frequently related to complications, postoperatively.	Noh 2022 [42]
97 esophageal carcinoma patients treated with CRT	NRS-2002	NRS-2002 score of 3 at diagnosis was related to better 2-year prognosis compared to an NRS-2002 score ≥ 4. NRS-2002 scoring at diagnosis was an independent risk factor for prognosis.	Wang 2018 [43]
Retrospective study with 143 patients with esophageal squamous cell carcinoma and adenocarcinoma followed for 20.8 months	NRI	NRI > 97.5 and PS = 0 were independently associated with overall survival times. Disease-free survival: NRI > 97.5 and PS = 0 were independent predictive factors.	Clavier 2014 [44]
Meta-analysis of 8 retrospective studies with 1460 esophageal squamous cell carcinoma patients	GNRI	Low GNRI was correlated with shorter overall and cancer-specific survival.	Fan 2022 [45]
Retrospective study on 107 esophageal carcinoma patients cured with neoadjuvant CRT and surgery	Weight status, Performance status Albumin	Reduced PS, difficulties in swallowing, weight decline prior to therapy, weight decrease > 5% throughout CRT, and serum albumin ≤ 35 g/L prior to or next to CRT implied shorter survival times. Serum albumin concentrations, nasogastric tube use, and weight decline prior to therapy were independently associated with overall survival. Serum albumin concentrations, along with nasogastric tube use next to CRT was associated with progression-free survival.	Zemanova 2012 [46]
Retrospective study on 74 patients with locally advanced esophageal carcinoma with adjacent organ invasion	Albumin and Hemoglobin	Younger age (<60 years) and hemoglobin levels above 13 g/dL were independently associated with favorable treatment efficiency. Elevated serum albumin (≥3.5 g/dL) prior to therapy was independently associated with favorable patients’ survival.	Hamai 2013 [47]
Retrospective study on 105 non-metastatic patients with a locally advanced esophageal carcinoma cured with definitive CRT	Albumin	Serum albumin > 35 g/L was independently associated with overall treatment efficiency. BMI > 18 Kg/m^2^, dysphagia Atkinson score < 2, dose of RT > 50 Grays, and CR to CRT were independently associated with favorable patients’ survival.	Di Fiore 2007 [48]
Retrospective study on 325 esophageal squamous cell carcinoma patients (256 surgical and 69 dCRT cases)	Sarcopenia	Sarcopenia substantially was associated with worse prognosis in patients with absence of lymph node metastasis, but not in patients presenting lymph involvement.	Harada 2015 [49]
Retrospective study on 42 patients, treated with a multimodal regimen of simultaneous neoadjuvant CRT, followed by surgery.	Adiponectin Serum albumin, and Cholesterol	In univariate analysis, elevated serum adiponectin was linked with poorer overall survival, while elevated serum albumin and cholesterol were associated with favorable overall survival. In multivariate analysis, only a tendency for negative serum adiponectin relationship with the overall survival was noted.	Zemanova 2014 [50]
Retrospective study on 100 patients with esophageal carcinoma cured with definitive chemoradiotherapy, preoperative chemoradiation, and definitive radiotherapy	PG-SGA BMI %Weight loss in 3 months Albumin Hemoglobin CRP GPS	PG-SGA score ≥ 9 was recognized as an independent predictor of radiation esophagitis.	Dong 2020 [51]
70 patients with esophageal and gastroesophageal junction carcinoma who underwent esophagectomy	GNRI Albumin Muscle mass %weight loss	Albumin and GNRI were decreased in patients developing severe complications compared to patients without postoperative complications. Major complications were related to weight decline and lower handgrip power. Albumin and poor muscle mass were considerably correlated with anastomotic leakage occurrence.	Lidoriki 2022 [52]
Retrospective study on 141 esophageal carcinoma patients undergoing neoadjuvant chemotherapy after radical esophagectomy	CONUT PNI	In multivariate analysis, malnourishment 14 days next to surgery according to CONUT. Lower PNI prior to surgery was identified as independent prognosticator of overall patients’ survival.	Hikage 2019 [53]
674 patients who underwent three-incision esophagectomy for esophageal carcinoma	CONUT	Malnutrition according to CONUT was an independent risk factor for severe, respiratory, and cardiovascular morbidities after surgical operation.	Horinouchi 2022 [54]

Song et al. (2017) followed up 202 patients with unresectable locally advanced esophageal carcinoma (stages 3 and 4) who were treated with definitive concurrent chemoradiotherapy. A Nutrition Risk Screening (NRS)-2002 score with a cut-off point of ≥3 was used to assess malnutrition at treatment initiation [41]. The NRS-2002 score was identified as an independent prognosticator for the response to chemoradiotherapy, overall patients’ survival, and progression-free patients’ survival [41]. The study by Noh et al. (2022), conducted on 274 patients (stages 1 to 3) undergone direct surgery for esophageal squamous cell carcinoma, showed that an elevated NRS 2002 scoring was associated with poorer prognosis and more complications postoperatively during a median follow-up of 55 months [42]. Moreover, an elevated NRS 2002 score was associated with frequent postoperative complications, especially pneumonia and anastomosis site leakage [42]. NRS-2002 was also evaluated in another study conducted on 97 individuals with esophageal carcinoma treated with chemoradiotherapy. Baseline NRS-2002 (cut-off point = 3) rates were independently associated with overall survival, but not with progression-free survival, organ metastasis-free survival, or local regional relapse-free survival [43].

Furthermore, Cox et al. (2016) found that a Nutritional Risk Index (NRI) of less than 100 was a predictor of malnutrition in esophageal carcinoma patients [24]. In fact, Clavier et al. (2014) evaluated several potential prognosticators for survival and causes of therapy interruption next to final chemoradiotherapy for esophageal carcinoma [44]. Among other factors, they examined the role of NRI in 143 individuals with esophageal squamous cell carcinomas and adenocarcinomas. An NRI higher than 97.5 and a Performance Status (PS) of zero (PS = 0) were independent prognosticators of 3 year and 5 year overall and disease-free survival with a median follow-up of 3 years and 5 years [44]. GNRI has also been assessed and was substantially related to shorter overall survival and cancer-specific survival amongst individuals with squamous cell carcinoma of the esophagus. In fact, a recent meta-analysis indicated that lower GNRI may be correlated with shorter overall and cancer-specific patient survival [45].

Other surveys have investigated the impact of PS, weight loss, and serum albumin levels as predictors of survival. Zemanova et al. (2012) in their retrospective study examined the influence of risk factors on overall survival and disease progression in 107 individuals with esophageal cancer who received neoadjuvant chemoradiotherapy and undergone surgery [46]. Among them, PS, body weight changes prior to and across chemoradiotherapy, difficulties in swallowing, dietary support, and serum albumin were included. Decreased PS, presence of difficulties in swallowing, demand for nasogastric tube use, more than average pretherapy weight decline, weight decrease above 5% across therapy, and serum albumin ≤ 35 g/L prior to or next to chemoradiotherapy implied a poor prognosis [46]. Serum albumin concentrations, nasogastric tube use, and pretherapy weight decline were independently associated with overall patient survival, while serum albumin levels next to chemoradiotherapy and nasogastric tube insertion were associated with disease development [46]. Additionally, a cross-sectional survey examined potential prognosticators in the monitoring of regional progressed esophageal carcinoma with close organ invasion and explored their effects in a sample of 74 individuals with esophageal carcinoma [47]. Older patients’ ages (≥60 years) and elevated pretherapy hemoglobin (≥13 g/dL) were independently associated with worse therapeutic outcomes, and elevated pretherapy serum albumin (≥3.5 g/dL) was independently associated with better prognosis [47]. Similarly, Di Fiore et al. (2007) documented that serum albumin > 35 g/L was independently associated with the final therapy response [48]. Moreover, BMI > 18 kg/m^2^, along with a dysphagia Atkinson score < 2, dose of radio treatment > 50 Grays, and final response to chemoradiotherapy were independently associated with favorable patient prognosis [48].

Furthermore, the impact of sarcopenia on the survival of individuals with esophageal squamous cell carcinoma followed by surgical resection or definitive CRT was examined [49]. It was found that sarcopenia was not considerably related to overall patient survival. Nevertheless, in patients with an absence of lymph node invasion, sarcopenia was related to shorter patient survival times, suggesting that it may function as a probable indicator for identifying patients who may present an unfavorable prognosis [49]. Zemanova et al. (2014) also evaluated the impact of nourishing, genetic, and inflammatory factors in the pathophysiology of esophageal squamous cell carcinoma in 42 male patients who received a multimodal treatment of simultaneous neoadjuvant chemoradiotherapy following surgical treatment [50]. In univariate analysis, elevated serum adiponectin was associated with worse overall patient survival, while elevated serum albumin and cholesterol were associated with favorable overall patient survival [50]. However, in multivariate analysis, only a trend of correlation for negative serum adiponectin association with the overall survival was observed [50].

As far as short-term outcomes and complications are concerned, further studies have been undertaken. A retrospective study was performed on 100 patients with esophageal cancer who were treated with definitive chemoradiotherapy, preoperative chemoradiation, and definitive radiotherapy [51]; 44% of the enrolled patients with a PG-SGA score ≥ 9 at baseline showed severe malnutrition, and 41% of patients developed grade ≥ 2 radiation esophagitis [51]. Multivariate analysis revealed that PG-SGA score ≥ 9 (*p* = 0.042) was the independent predictor of radiation esophagitis [51]. Regarding postoperative complications, studies have shown that greater body weight loss, low albumin levels, and low GNRI were predictors for major complications [52], while high NRS-2002 score [42] and low PNI [34] were also associated with frequent postoperative complications. In fact, in the study of Lidoriki et al., 52.9% of the patients developed postoperative complications and both albumin and GNRI levels were lower in patients who developed major complications compared to patients who did not develop postoperative complications [52]. Major complications were associated with a higher percentage of weight loss and with low handgrip strength, while albumin and low muscle mass were significantly associated with anastomotic leakage occurrence [52]. Furthermore, Hikage et al. (2019) retrospectively evaluated 141 esophageal cancer patients who were treated with neoadjuvant chemotherapy postradical esophagectomy and found that, based on the CONU score, malnutrition occurred only from 14 days after surgery in most cases. According to PNI, the ratio of malnutrition increased gradually from presurgery to 14 days after surgery [53]. A multivariable analysis of independent prognostic factors predicting survival identified malnutrition 14 days after surgery with the CONUT score and a low PNI before surgery, invasion depth of the primary lesion, and node metastasis. [53]. Further studies have also highlighted the prognostic value of CONUT for short-term postoperative complications after esophagectomy [30,54]. Notably, in the study of Horinouchi et al., a total of 674 patients who underwent esophagectomy (296) and minimally invasive esophagectomy (378) were analyzed [54]; 32 patients of the esophagectomy group and 16 of the minimally invasive esophagectomy group were classified as having moderate and severe malnutrition, respectively. Moderate and severe malnutrition was significantly associated with a low BMI, poor performance status, poor American Society of Anesthesiologists physical status, advanced cancer stage, and frequent preoperative treatment [54]. These patients also showed considerably more frequent morbidities of grade ≥ IIIb based on the Clavien–Dindo classification (CDc), respiratory, and cardiovascular morbidities after esophagectomy [54]. Moreover, moderate and severe malnutrition in CONUT was an independent risk factor for morbidity of CDc ≥ IIIb, respiratory, and cardiovascular morbidities [54]. Thus, preoperative malnutrition in CONUT reflected diverse disadvantageous clinical factors and may be considered a predictor of worse short-term outcomes after esophagectomy; however, it had no value in minimally invasive esophagectomy [54].

### 3.2. Pharyngeal Cancer

Several nutritional assessment tools have been used to explore the prognostic role of nourishing state on pharyngeal cancers, as described in Table 2. The recent retrospective study by Wu et al. (2022) highlighted the fact that several markers of nutritional status and assessment tools have prognostic value for pharyngeal cancers [55]. More to the point, 319 pharyngeal cancer patients were recruited with a diagnosis of nasopharyngeal carcinoma, oropharyngeal carcinoma, and hypopharyngeal carcinoma [55]. Multiple nutritional markers, including BMI, hemoglobin, albumin, PNI, NRI and hemoglobin, albumin, lymphocyte, and platelet (HALP) score were important predictors for pharyngeal cancers in univariate regression analysis [55]. In multivariate analysis, the HALP score remained an independent factor for overall survival after adjusting for gender, age, cancer site, clinical stage, and BMI. The PNI was the most important independent factor for overall survival and cancer-specific survival [55]. Accordingly, a meta-analysis of 10 studies containing 4511 patients with nasopharyngeal carcinoma showed that patients with a low PNI had worse overall survival, distant metastasis-free survival, progression-free survival, and locoregional recurrence-free survival. A subgroup analysis also showed that the low PNI was still a significant prognostic factor for overall survival and distant metastasis-free survival [56].

Thus, reduced PNI was recognized as a substantial predictor of shorter overall survival, organ metastasis-free survival, progression-free survival, and locoregional relapse-free survival in individuals with nasopharyngeal cancer [56], and also a prognostic factor of overall survival and relapse-free survival in individuals with esophageal squamous cell cancer [57]. Other recent studies have agreed with these findings. In fact, Topkan et al. (2021) recently showed that low baseline PNI (cut-off point at 51) was an independent prognosticator for overall survival, cancer-specific survival, locoregional progression-free survival, organ metastasis-free survival, and progression-free survival in 154 individuals with locoregionally progressed nasopharyngeal cancer treated simultaneously with chemoradiotherapy [58]. Notably, the negative effect of the reduced PNI remained significant for a follow-up period of 10 years [58]. Additionally, a PNI lower than 51 was substantially related to greater levels of weight decrease >5% during the previous 6 months compared to the PNI < 51 group [58]. Moreover, PNI was evaluated in a recent survey by Küçükarda et al. (2022), in 107 nonmetastatic nasopharyngeal cancer patients, who were assessed before and after treatment [59]. PNI at both time periods was identified as an independent prognosticator for overall patient survival, while pretreatment PNI with a cut-off point of ≤50.65 was associated with worse locoregional relapse-free survival, and organ metastasis-free survival [59]. Moreover, after chemoradiotherapy for advanced cancers (stages 3 and 4) of the oral cavity, oropharynx, and hypopharynx, low PNI was significantly associated with toxicity and toxic death, as well as with T categorization and progressive histopathological staging [59]. Patients presenting decreased PNI exhibited a lower probability of accepting concurrent chemoradiotherapy and needed also more frequent tube feeding support [60]. Regarding hypopharyngeal squamous cell carcinoma, high PNI at a cut-off point of 52 was identified as an independent prognosticator for better overall, progression-free, locoregional relapse-free survival and organ metastasis-free survival in 123 patients at all stages of the disease, of whom 16.3% were at stage 3 and 69.1% at stage 4 [61].

**Table 2:** Studies regarding pharyngeal cancers and the prognostic role of nutritional status.

Characteristics of Individuals with Pharyngeal Carcinoma	Assessment Tool	Results	Author, Date
319 patients with nasopharyngeal, oropharyngeal and hypopharyngeal cancer	BMI, hemoglobin, albumin, PNI, NRI, HALP	HALP scoring was independently associated with overall survival after adjustment for sex, age, tumor site, histopathological stage, and BMI. In multivariate analysis, PNI was identified as the most essential indicator for overall and cancer-specific survival.	Wu 2022 [55]
Meta-analysis of 10 studies with 4511 patients with nasopharyngeal carcinoma	PNI	Patients with decreased PNI exhibited a worse overall, organ metastasis-free, progression-free, and locoregional relapse-free survival. Subgroup analysis: PNI was significantly associated with overall and distant metastasis-free survival.	Tu 2020 [56]
154 patients presenting locoregionally advanced nasopharyngeal cancer treated with concurrent chemoradiotherapy	PNI	↓ baseline PNI (cut-off point = 51) is an independent prognosticator for overall, cancer-specific, locoregional progression-free, organ metastasis-free, and progression-free survival.	Topkan 2021 [58]
107 nonmetastatic nasopharyngeal carcinoma patients	PNI	Pre- and post-treatment PNI were independent predictors for overall survival.	Küçükarda 2022 [59]
143 patients presenting stage III, IVA, and IVB pharyngeal cancers who were treated with concurrent chemoradiotherapy	PNI	Patients with lower PNI exhibited elevated likelihoods of grade 3/4 hematological toxicities, sepsis, and toxic death. Patients with lower PNI were less probable to tolerate concurrent chemoradiotherapy, even when they were treated with a considerably lower dosage of cisplatin, showing a decreased completion rate of planned radiotherapy, or a longer overall radiotherapy treatment time.	Chang 2018 [60]
123 hypopharyngeal squamous cell carcinoma patients treated with radical surgery	PNI	Higher PNI was independently associated with greater overall, progression-free, locoregional relapse-free, and organ metastasis-free survival preoperatively.	Ye 2018 [61]
359 newly diagnosed nasopharyngeal cancer patients undergoing intensity-modulated radiation therapy	CNI	CNI decreased after therapy. CNI was independently associated with overall survival.	Deng 2019 [62]
Retrospective study with 309 older nasopharyngeal carcinoma patients	CNI	CNI was independently associated with overall and disease-free survival. Reduced CNI was associated with unfavorable overall and disease-free survival.	Duan 2021 [63]
187 nasopharyngeal cancer patients who had normal nutrition before treatment	modified Nutrition Index (m-NI)	Severe nutritional impairment was an independent prognosticator for overall survival, being identified as a significant risk indicator of grade ≥ 2 oral mucositis.	Su 2020 [64]
228 nasopharyngeal cancer patients with NPC treated with intensity-modulated radiotherapy	modified Nutrition Index	m-NI ≤ 6 was identified as a significant indicator for xerostomia, oral mucositis, dysgeusia, and dysphagia. Radiation-induced acute toxicities of malnourished individuals were considerably greater compared to those of individuals with physiological nutritional status.	Song 2023 [65]
323 patients with nasopharyngeal carcinoma undergoing intensity-modulated radiotherapy	modified Nutrition Index	The 1, 3, and 5 year overall survival times between malnourishment and physiological nutritional status groups assessed by m-NI were 93.0% vs. 96.9%, 76.4% vs. 82.8%, and 61.8% vs. 77.1%, respectively. m-NI was independently associated with overall survival.	Hong 2017 [66]
3232 nasopharyngeal carcinoma patients from a big-data database	NRS-2002	NRS2002 ≤ 3 vs. >3 had significantly different 5 year disease-free, overall, distant metastasis-free, and locoregional relapse-free survival.	Peng 2018 [67]
59 patients presenting clinical stage III and IV hypopharyngeal squamous cell carcinoma underwent pharyngo–laryngo–cervical esophagectomy with definitive tracheostomy followed by free jejunal graft reconstruction.	GPS NLR	↑ GPS (1 or 2) and ↑ NLR (≥5) were independent unfavorable prognosticators for 5 year overall survival.	Ikeguchi 2016 [68]
Prospective survey of 512 patients with nasopharyngeal carcinoma undergone radical RT	Weight status (ideal body weight, IBW) Albumin	Prior to radiotherapy, IBW% < 90% was related to worse overall and organ metastasis-free survival. Albumin ≤ 43.0 g/L was linked to worse overall and metastasis-free survival.	Li 2014 [69]
53 patients presenting locally advanced hypopharyngeal carcinoma (stages 3 and 4) assigned to an induction chemotherapy (ICT)-based larynx preservation program without prophylactic feeding-tube placement	Weight loss	Maximum weight loss was considerably correlated with a greater probability of enteral tube feeding during treatment and a higher likelihood of complications during radiotherapy.	Bozec 2016 [70]

CNI, which considers BMI, typical body weight, hemoglobin, and albumin concentrations, as well as total lymphocyte amount, has been assessed in 359 newly diagnosed individuals with nasopharyngeal cancer treated with intensity-modulated radiation therapy (IMRT) [62]. This study clearly showed that decreased CNI was an independent prognosticator of overall survival [62]. The CNI was relatively modest in patients with the III-IV clinical tumor stage as well as in patients receiving induction chemotherapy in combination with simultaneous chemotherapy. After IMRT, reduced CNI scoring was linked with a worse quality of life [62]. Similarly, in stages 3 and 4, older individuals with nasopharyngeal carcinoma underwent radiotherapy, CNI was used to determine the prognostic role of nutritional status, and it was found that it was independently associated with overall and disease-free survival [63]. In the same survey, both PNI and NRI were also assessed with similar results [63].

Su et al. (2020) explored the prognostic role of the modified Nutrition Index (m-NI) on therapy toxicity and survival in 187 individuals with nasopharyngeal cancer who had normal nutrition before treatment. Severe nutritional impairment during IMRT, which was assessed as a reduction in m-NI score of ≥50%, was independently associated with overall patients’ survival and oral mucositis [64]. Similarly, the recent survey by Song et al. (2023) demonstrated that malnutrition before radiotherapy, assessed via m-NI, was a predictor of short-term clinical complications such as severe dysgeusia, oral mucositis, dysphagia, and xerostomia next to radiotherapy for nasopharyngeal cancer, which, in turn, worsen patients’ nutritional statuses [65]. Regarding overall survival, the follow-up survey of Hong et al. (2017) on 323 individuals with nasopharyngeal carcinoma who underwent intensity-modulated radiotherapy indicated that the m-NI was a substantial prognosticator for 1, 3, and 5 year overall survival in this patient group [66].

Furthermore, NRS-2002 was evaluated by Peng et al. (2018) in nasopharyngeal carcinoma patients, utilizing a large-data intelligence database platform and detected 3232 patients [67]. Patients presenting NRS2002 ≤ 3 vs. >3 exhibited considerably different 5 year disease-free survival, overall survival, organ metastasis-free survival, and locoregional relapse-free survival [67]. This survey used a different cut-off point for the NRS-2002. In fact, the European Society for Clinical Nutrition and Metabolism (ESPEN) recommended a ≥3 score to be the cut-off for nutritional impairment, and not >3 [67]. Other tools that have been utilized are the Glasgow Prognostic Score (GPS) and the Neutrophil–Lymphocyte Ratio (NLR) in locally advanced hypopharyngeal squamous cell carcinoma patients undergone pharyngo–laryngo–cervical esophagectomy, reconstructed by jejunal graft [68]. Poor PS according to GPS, and high NLR (cut-off point at ≥5) were independently associated with shorter survival times, while elevated preoperative GPS was identified as a significant risk indicator concerning postoperative complications [68].

Weight status (percentage of ideal body weight) and albumin levels were evaluated in a group of 512 patients with nasopharyngeal carcinoma receiving radiotherapy [69]. Both parameters were identified as independent prognosticators for overall patient survival, while pretreatment body weight at <90% of the ideal body weight was linked with worse overall and distance metastasis-free survival [69]. However, Bozec et al. (2015) failed to find significant relationships between nourishment state and clinical outcomes in regionally progressed (stages 3 and 4) cancer patients, of whom 11,3% had lost significant weight (>10%) and 32% needed enteral nutrition [70]. Only WHO PS and minimum weight loss were identified as significant independent prognostic factors for complications during radiotherapy [70]. The explanation for this finding may be the rather reduced incidence of malnourishment in this group under study, as pretreatment malnutrition rates in this patient group were 19–45% [71].

## 4. Conclusions

Malnourishment in individuals with cancer constitutes one of the major prominent factors in the progression and mortality in such patients, and cancer cachexia significantly increases the risk of mortality. To slow down the prevalence of malnourishment, it is strongly recommended to develop an effective and systematic nutritional intervention, which should be personalized according to the specific characteristics of cancer patients. For this purpose, validated and accurate nutritional assessment tools and specific indicators have been developed to determine the specific patient’s condition. At this time, there are currently several nutritional assessment tools, which are used independently of the type of cancer. This is a significant and remarkable gap in the international literature since each type of cancer has different histopathological disease progressions and prognostic characteristics. Notably, this is very noticeable in our study on esophageal and pharyngeal cancers where different nutritional assessment tools were used even in the case of only one type of cancer.

Regarding esophageal cancer, nourishment state has been identified as a substantial indicator of patients’ survival and short-term treatment complications. Several tools have been evaluated around the world, with CONUT, PNI, PG-SGA, and NRS-2002 being more common in the literature, while albumin is also frequently evaluated regarding clinical outcomes. Recent meta-analysis studies have also indicated that sarcopenia could be utilized as a significant indicator of worse survival [72], while GNRI and PNI could also be utilized as efficient indicators of survival in esophageal squamous cell carcinoma [45,57].

As far as pharyngeal cancers are concerned, fewer studies are currently available. PNI has been evaluated and its significance as a potential factor for shorter overall patient survival, organ metastasis-free survival, progression-free survival, and locoregional relapse-free survival has been highlighted. CNI has also been investigated with positive results, as well as NRS 2002 and GPS. Regarding body-weight status, most studies do agree that body-weight loss may be considered a prognosticator for survival, which is necessary for nutritional support across the therapy.

It should be noticed that most studies are observational and retrospective, and are comprised mainly of men, with women being the minority of the study populations. In this aspect, recall bias should be taken into consideration, while the causality effect cannot be supported. Hence, further prospective, large-scale, well-designed clinical surveys that investigate the prognostic role of nourishment state as well as studies that evaluate the impact of improving the nutritional status on survival and clinical outcomes are strongly recommended. Last but not least, the lack of studies that focus on female patients is of great importance, as gender differences may be present [73].

The currently available studies in these patient groups regarding the prognostic impact of nutritional status on disease progression and survival outcomes strongly highlight the significance of maintaining a good nourishment state and/or intervening with the aim of supporting patients and make better their nutritional status [74,75,76]. The nutritional assessment tools that have been utilized and evaluated are easily utilized and are currently used in routine clinical practice. Nevertheless, there is a strong demand to investigate which nutritional assessment tool is more suitable and effective for each cancer type separately, and, thus, future clinical studies are recommended to systematically focus on this direction.

## Data Availability

The data of the study are available upon request to the corresponding author.
